# Application of 3D-printed porous prosthesis for the reconstruction of infectious bone defect with concomitant severe soft tissue lesion: a case series of 13 cases

**DOI:** 10.1186/s12891-024-08248-6

**Published:** 2024-12-30

**Authors:** Zhuo Chen, Yiyuan Yang, Bingchuan Liu, Xingcai Li, Yun Tian

**Affiliations:** Department of Orthopedics, Peking University Third Hospital, No.49, North Garden Rd, HaiDian District, Beijing, 100191 China

**Keywords:** 3D Printed prosthesis, Infectious bone defects, Tibial bone defect, Soft tissue lesion, Chronic osteomyelitis, Flap transfer

## Abstract

**Background:**

Treating infectious bone defects combined with large soft-tissue lesions poses significant clinical challenges. Herein, we introduced a modified two-stage treatment approach involving the implantation of 3D-printed prostheses and flap repair to treat large segmental infectious tibial bone defects.

**Method:**

We conducted a retrospective study of 13 patients treated at our center between April 2018 and March 2022 for tibial infections owing to posttraumatic infection and chronic osteomyelitis combined with soft tissue defects. The average defect length was 14.0 cm (range, 5.7–22.9 cm). The flap area ranged from 14 × 5 to 15 × 8 + 25 × 15 cm. Sural neurocutaneous, lesser saphenous neurocutaneous, and local fasciocutaneous flaps were used to repair the skin defects. In the second stage, 3D-printed prostheses were designed and implanted. Union rate, complications, and functional outcomes were assessed at the final follow-up.

**Result:**

The average follow-up period was 31.1 months (range, 17–47 months), with an average interval of 208.1 days (range, 139–359 days) between the two stages. According to our criteria, 7 of the 13 patients achieved radiographic healing without intervention. Two patients developed prosthesis-related complications and underwent revision surgery. Two patients experienced recurrent infections leading to prosthesis removal and debridement surgery, with the infection ultimately eradicated in one and the other undergoing amputation. Three patients experienced noninfectious flap-related complications, however, all eventually healed through surgical intervention.

**Conclusion:**

The use of 3D-printed porous titanium prostheses combined with flap soft-tissue repair for the treatment of infectious tibial bone defects did not increase the rate of infection recurrence and provided good functional recovery, offering more options for the treatment of infectious bone defects.

## Introduction

Treatment of infectious bone defects is a constant challenge in orthopedics. Infection causes severe damage to the bone and soft tissue, especially in patients with chronic sinus tracts, which are highly destructive. Patients with these conditions often experience a prolonged course of illness and undergo multiple surgeries. Radical debridement is essential to ensure the eradication of the infection [1, 2]. However, debridement requires the removal of a large area of necrotic bone, infected soft tissue, and skin, leading to extensive bone and soft tissue defects [3]. Tibial defects are more challenging to treat than femoral defects. Poorer blood supply and thinner soft tissue of the tibia lead to higher recurrence rates and earlier recurrences of infections [4, 5]. The repeated alleviation and recurrence of infection, chronic inflammation, scar hyperplasia, and dense fibrosis of soft tissues lead to the loss of normal tissue planes, making soft tissue reconstruction burdensome [6, 7].

Various treatment methods with good clinical outcomes on the reconstruction of lower limb bone and soft tissue defects, such as Ilizarov [1, 7–11], Masquelet [12, 13], and autologous fibula grafts combined with flap transplants [14], have been reported. Meanwhile, traditionally, internal fixation as a foreign body could potentially stimulate biofilm formation [15] and increase the presence of recurrent infections, necessitating cautious use of prostheses and internal fixation in infection cases. However, with the development of 3D printing technology and clinical experience, internal fixation is not contraindicated after thorough debridement [4].

Furthermore, 3D-printing technology allows the production of prostheses that precisely match the anatomical morphology of the defect site, effectively correcting limb length and angular deformities and promptly restoring mechanical stability. Adjusting the porosity of a prosthesis minimizes the stress shielding effect. Additionally, good bone integration has been observed in animal experiments and case studies, which provides the foundation for the long-term stability and efficacy of prostheses [16–18]. Thus, the purpose of this study is to evaluate the clinical outcomes of 3D-printed prostheses in treating extensive tibial bone and soft tissue defects caused by infection. To the best of our knowledge, no reported cases of the application of 3D-printed prostheses combined with flap transplantation for treating infectious tibial bone defects exist. The 3D-printed prostheses may provide new insights into the treatment of infectious bone defects by achieving effective bone integration, restoring mechanical stability, and reducing the need for secondary surgeries.

## Materials and methods

### Patients

We retrospectively studied 13 patients who were treated at our center between April 2018 and March 2022. The inclusion criteria were as follows: (1) patients aged 18 years or older, (2) patients with infectious bone defects accompanied by soft tissue defects, and (3) patients with a minimum follow-up duration of one year. All patients provided written informed consent for their participation. The study was approved by the Peking University Third Hospital Medical Science Research Ethics Committee (Beijing, China). Patients with (1) defects owing to tumor resection, (2) defects involving joints, (3) Charcot joints and (4) those with less than one year of follow-up were excluded. All patients underwent a two-stage treatment. The first stage involved debridement, polymethylmethacrylate (PMMA) spacer insertion, and flap transplantation. The second stage involved the implantation of a 3D-printed prosthesis. Table 1 presents the demographic data, bone defect size, soft tissue defect size, defect location, debridement time, and comorbidities.

**Table 1:** Demographic characteristics of patients studied

Case	Location	Microorganism	BMI (kg/m^2^)	Comorbidity	Bone defect length (cm)	Soft Tissue defect(cm)	Times of debridement	Type of transferred flap	Size of flap (cm)	Duration between two stages (d)	Follow-up (mo)
1	distal	NA	22.6	ankle limitation	14	10*5	2	SNF	15*8	210	26
2	Proximal, middle, distal	NA	18.4	Ankle limitation, discrepancy	22.9	30*15	3	Local fasciocutaneous flaps	15*8 + 25*15	154	40
3	Distal	Acinetobacter baumannii, Enterobacter cloacae	22.3	coronary heart disease	5.7	18*5	3	SNF	20*6	177	27
4	Proximal	pseudomonas aeruginosa	21.6	Superficial infection	10.8	12*5	7	LSNF	18*7	159	43
5	Distal	enterococcus faecalis, Acinetobacter baumannii, Staphylococcus haemolyticus, Acinetobacter pittii	28.4	Diabetes,Superficial infection	10.1	10*5	10	SNF	14*5	139	38
6	Middle, distal	Klebsiella aerogenes	24.5	Superficial infection	20.5	20*5	4	SNF	24*7	280	30
7	Middle	Staphylococcus aureus; Pseudomonas aeruginosa	28.7	NA	12.4	15*8	5	SNF	20*8	186	18
8	Proximal, Middle, Distal	Enterobacter cloacae	30.`	Diabetes, sinus, Ankle limitation	22.2	20*7	7	SNF	20*8	253	18
9	Distal	Staphylococcus aureus;	24.9	sinus	13.9	25*5	13	SNF	27*7	253	20
10	Distal	NA	26.2	Diabetes, depression	11.2	15*5	2	SNF	18*7.5	144	20
11	Distal	methicillin-resistant staphylococcus	28.4	Hypertension, peroneal nerve injury	12.5	15*7	6	SNF	19*10	249	13
12	Middle	staphylococcus caprae	23.1	Ankle limitation	21.5	20*7	9	SNF	22*10	359	16
13	Proximal	alcaligenes xylosoxidans,klebsiella pneumoniae,enterococcus faecium	27.1	Osteoporosis, peroneal nerve injury	7	18*8		Local fasciocutaneous flaps	20*10	142	34

*SNF* Sural neurocutaneous flap, *LSNF* Lesser saphenous neurocutaneous flap

### Surgical technique (Fig. 1)

**Fig. 1:**
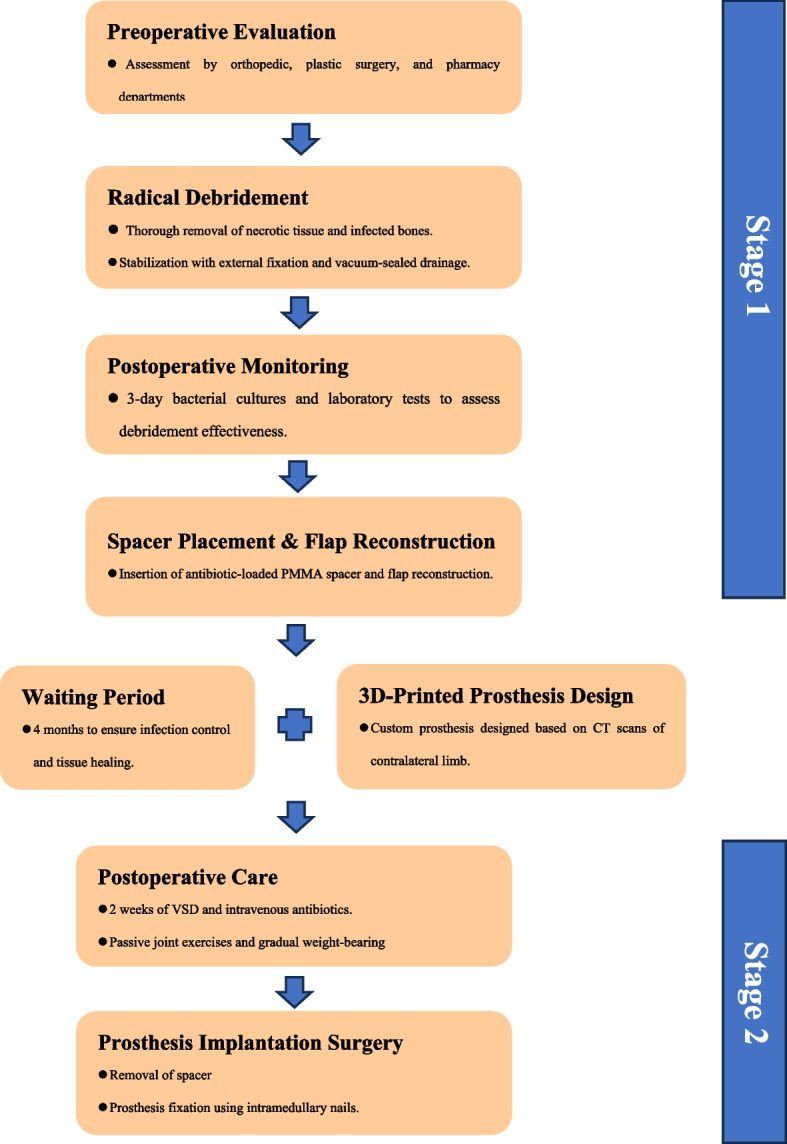
Workflow for 2 stages treatment

#### First stage

The treatment plan was developed collaboratively by a multidisciplinary team, including orthopedic, plastic surgery, and pharmacy departments. The surgeries were performed by experienced orthopedic and plastic surgeons with over 15 years of clinical experience. Pre-operative cultures were taken when patients presented with sinus tracts, purulent exudate, or non-healing wounds. The patients were positioned appropriately on a traction table to correct discrepancies in limb alignment and angular deformities, and to ensure stable position. During debridement, necrotic soft tissue, scars, skin, and infectious bone were thoroughly removed until bleeding from the margin of the cortical bone was observed. The proximal and distal bone interfaces were trimmed to planes perpendicular to the long axis of the bone shaft to prepare for the subsequent prosthesis design and placement. The wounds were thoroughly irrigated with a large volume of hydrogen peroxide, povidone-iodine, and saline. Necrotic tissue, drainage fluid, and infected bones were collected and subjected to bacterial culture in more than five cases. Subsequently, traction was applied to restore the limb length as much as possible, and an appropriate external fixator was used for stabilization. The wounds were covered with vacuum-sealed drainage (VSD), and intravenous antibiotic was administered empirically to combat the infection, with dose adjustments based on bacterial culture and sensitivity results. The drainage fluid was retained for three days postoperatively for bacterial culture to assess the effectiveness of debridement. If any culture was positive, the debridement procedure was repeated the following week. Orthopedic and plastic surgeons performed bone and soft tissue reconstruction surgery immediately after three consecutive cultures were negative, and the results of white blood cell count (WBC), C-reactive protein levels (CRP), and erythrocyte sedimentation rates (ESR) confirmed the absence of clinical signs of infection. An orthopedic surgeon inserted a PMMA spacer consisting of 40 g of PMMA bone cement and 2 g of vancomycin into the defect area. The spacer was slightly larger than the bone defect to facilitate subsequent prosthesis placement and further restoration of limb length. The plastic surgeon subsequently designed the flap, with a preference for using a reverse sural neurocutaneous flap (SNF). The edges of the flap were positioned as far as possible from the debridement area. Based on the location of the rotation point, the design of the flap was dependent on the course of the superficial nerve, size of the defect, and requisite length of the pedicle. The edge of the flap was 2–3 cm larger than the defect area to counteract skin contractions and provide space for prosthesis placement. Dissection of the flap was performed in layers from the lateral aspect of the leg, preserving and protecting the fascial pedicle. Subsequently, the flap was rotated to cover the wound. Donor site coverage was achieved using a thick abdominal skin graft, and the VSD was applied to all incision sites to ensure cleanliness and enhance adhesion. Local fascial flaps were used for tissue reconstruction when SNFs were not feasible.

#### Prosthesis design

**Fig. 2:**
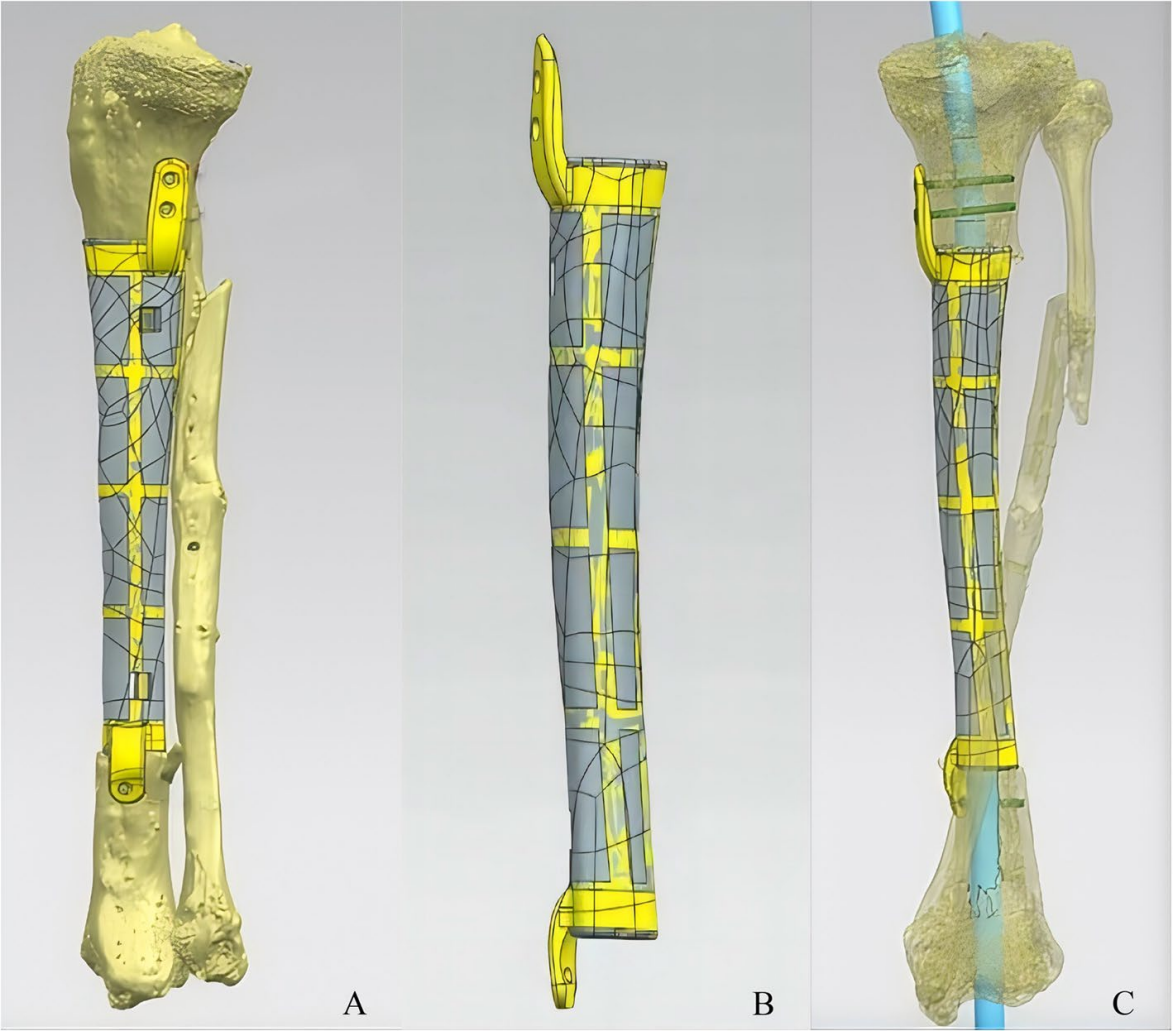
The design drawings of the 3D printed prosthesis. All prostheses were designed to be hollow to accommodate intramedullary nails and one-piece lateral plate were designed to ensure stability

According to the workflow [19], all patients were required to undergo full-length computed tomography scans of both lower limbs following completion of the first-stage treatment, and the prostheses were designed using 3D-printing based on the contralateral limb. The prostheses design process was completed using the Mimics software (Mimics 21.0; Materialize, Leuven, Belgium) and was produced by AK Medical Holding Ltd. The prostheses were made of titanium alloy (Ti6Al4V) and consisted of a regular icosahedron microporous unit structure with a porosity of 60–80% and a pore diameter of 625 ± 70 μm based on research and our previous animal experimental study [18, 20–22]. All prostheses were designed to be hollow to accommodate intramedullary nails, with the hollow diameter of the prosthesis designed to be 1–1.5 mm wider than the planned intramedullary nail diameter. Depending on the defect location, when the defect involved the metaphysis, a one-piece lateral plate was designed to ensure stability and torsional resistance before new bone ingrowth, thereby providing biomechanical stability. The prosthesis had a 3 mm × 3 mm drug-loading window for vancomycin loading. When the residual bone was insufficient to accommodate the three intramedullary nails, a one-piece intramedullary nail prosthesis was used. Subsequently, the lateral plate was fixed with more than three locking screws to ensure the initial stability (Fig. 2).

#### Second stage

Patients were discharged and observed for more than 4 months after the first stage of treatment, and oral second-generation cephalosporins were administered during the waiting period. Patients met the following criteria before the second stage treatment was commenced: 1. Laboratory tests, including WBC, CRP, and ESR rate, revealed no abnormalities; 2. The flap and skin graft areas showed no signs of redness, swelling, heat, pain, or sinus formation; 3. No evidence of systemic infection. Plastic surgeons designed the surgical incision based on flap healing conditions before surgery. Pre-operative vascular status was evaluated using Doppler ultrasound and CT angiography. The flap was carefully elevated during surgery, and the bone cement was removed. If a discrepancy in the limb length remained constant, appropriate traction was applied. The prosthesis was fixed using intramedullary nails without the need for bone grafting. Based on our experience, the intramedullary nails initially disperse more stress; therefore, three locking screws were required at both ends of a prosthesis to ensure stability. Similarly, when a one-piece prosthesis was used, three or more locking screws were required for fixation. 2 g vancomycin was placed into the drug-loaded hollows. Wounds were closed in layers, and drainage was placed, with the incision covered with VSD and subjected to two weeks of negative pressure suction to reduce soft tissue tension caused by surgical trauma. Postoperatively, flap color and blood supply changes were closely monitored, and broad-spectrum sensitive antibiotics were administered intravenously as routine measures. Wound drainage was removed 48 h postoperatively if the drainage volume was < 20 ml. Following drainage and VSD removal, the patients were allowed to perform passive joint exercises in bed and gradually progressed to partial weight-bearing training with crutches, eventually achieving full weight-bearing within two months.

### Outcome parameters

Patients were required to return for follow-up at 1, 3, 6, and 12 months and every year thereafter. Full-length radiographic imaging of the lower limbs in the anteroposterior and lateral views were asked at each follow-up. The outcomes assessed included the infection eradication rate, time to bone healing, implant complications, and postoperative complications. Owing to the inability to directly observe bone ingrowth, the radiographic union was defined as follows: (1) prosthetic dislocation < 2 mm, (2) continuous bone formation across the bone interface and extent of prosthesis (proximal or distal), (3) no remaining infection, and (4) no periprosthetic translucent band on radiography [22].

## Results

**Fig. 3:**
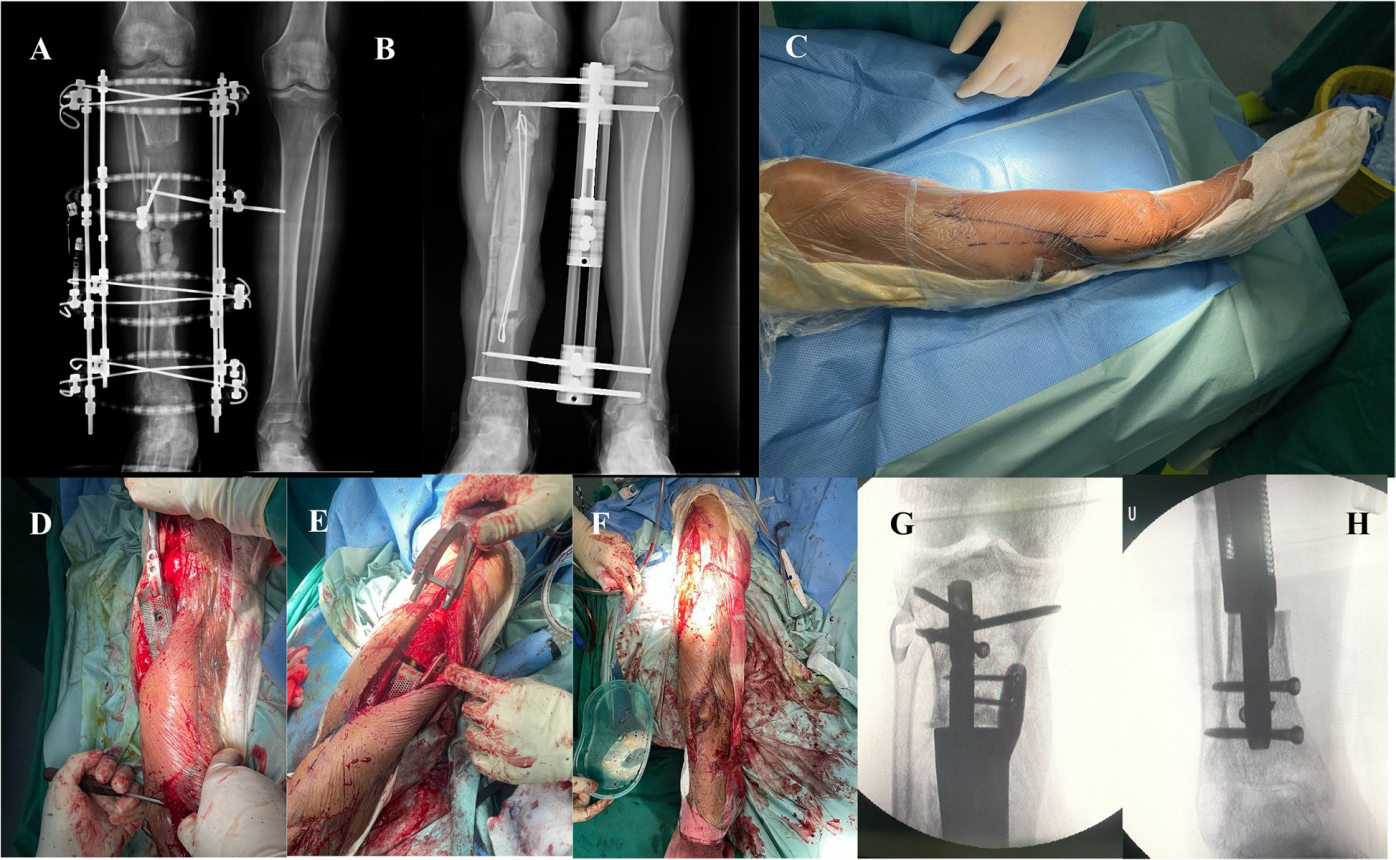
**A** A 51-year-old male suffered bone nonunion during the treatment in an external fixator for 2 months. **B** After radical debridement, a PMMA spacer was inserted, and an external fixator was placed. **C** Plastic surgeons designed the surgical incision based on flap healing conditions. **D** The double incision was used due to the flap condition, the prosthesis was implanted after the bone cement was removed. **E** The prosthesis was fixed using intramedullary nails. **F** The flaps was designed with a diameter 2–3 cm larger than the defect area, which provided sufficient space for the 3D-printed prosthesis. **G**, **H** Three locking screws were required at both ends of a intramedullary nail to ensure stability

The study included 12 males and 1 female, with a mean age of 48.7 years (range, 34–76 years). Before prosthesis implantation, all patients underwent an average of 5.9 (2–13) debridement surgeries. The average length of the defect was 14.0 cm (range, 5.7–22.9 cm). The flap area ranged from 14 × 5 cm to 15 × 8 + 25 × 15 cm. The average follow-up period was 31.1 months (range, 17–47 months), and the average interval between the two stages was 208.1 days (range, 139–359 days). Ten SNFs, two local fasciocutaneous flaps, and one lesser saphenous neurocutaneous flap were transferred to resurface the soft tissue defects. Two patients (case 2 and case 10) underwent intraoperative flap transplantation due to incomplete wound closure after prosthesis placement (Fig. 3).

**Fig. 4:**
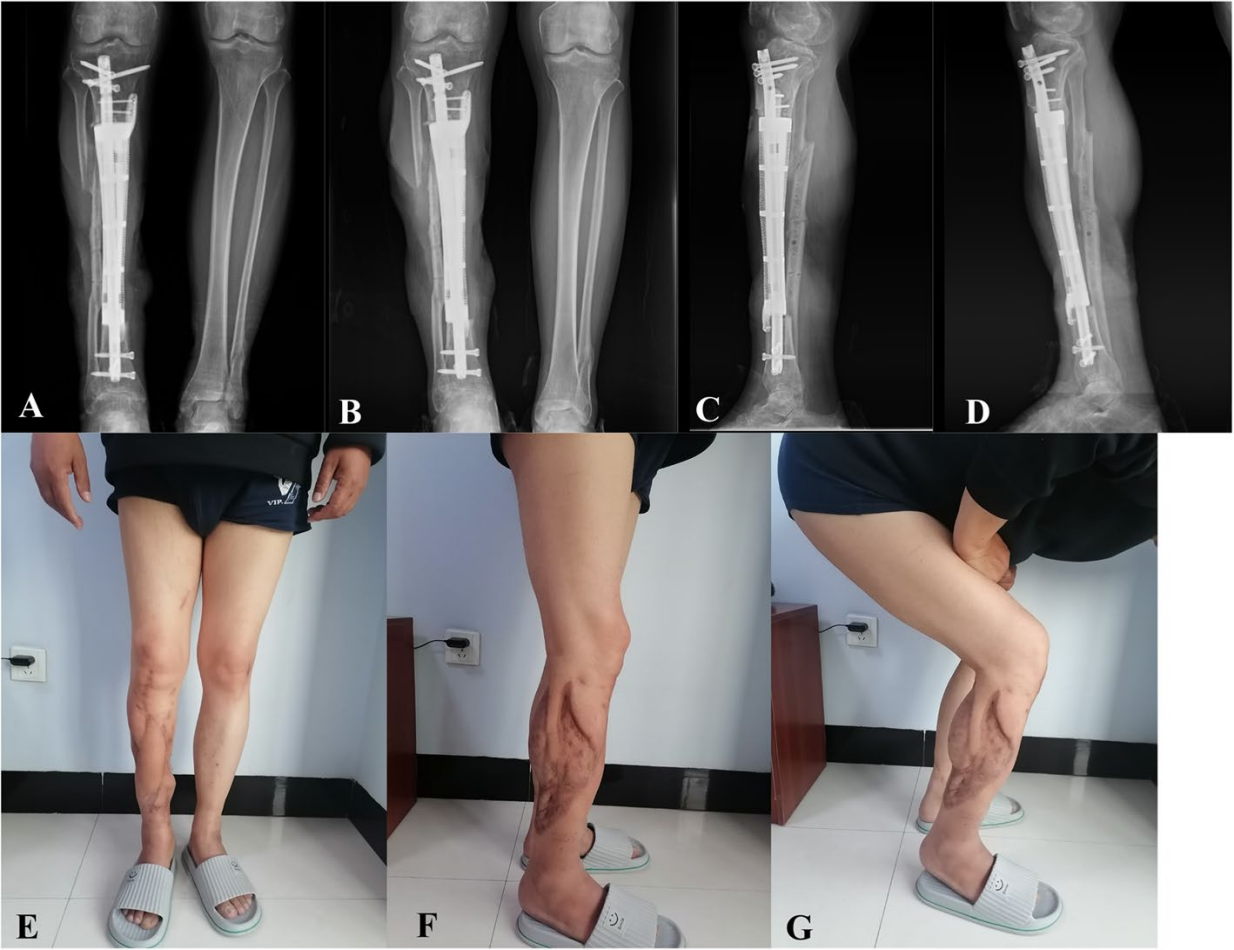
**A**, **B** Anteroposterior radiographs of the immediate postoperative (**A**) and 12-month follow up (**B**), showing the stability of the reconstruction. **C**, **D** Lateral radiographs of the immediate postoperative (**C**) and 12-month follow up (**D**). Continuous callus formation across the bone interface could be observed on the proximal. **E**,**F**,**G** The wound heal well with satisfactory appearance and could satisfy the needs of daily social work

**Fig. 5:**
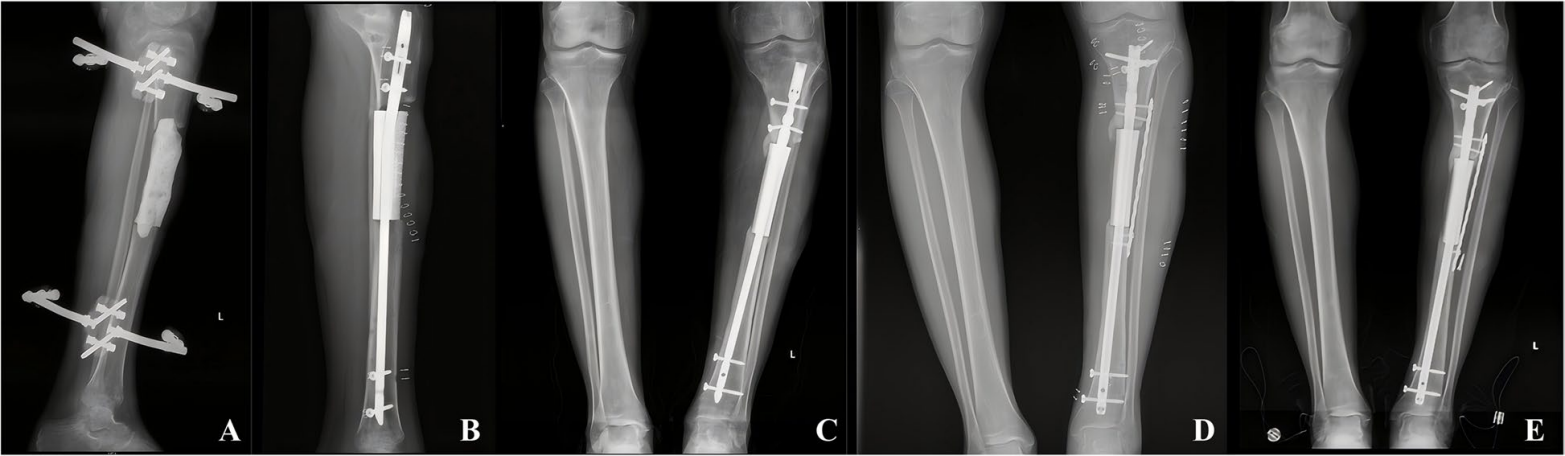
Case 4. A 37-year-old patient who suffered prosthesis displacement and limb deformity due to an intramedullary nail locking screw fracture. **A**, **B** Lateral radiographs of the first treatment. **C** The patient complained of pain at 6-month follow-up, angular displacement of the prosthesis and locking screw fracture can be observed on X-ray. **D** Revision surgery was performed with intramedullary nail replacement and internal plate fixation. **E** Showing the stability of the reconstruction at 3-year follow-up after revision surgery

According to our criteria, 7 of the 13 patients achieved radiographic union without intervention (Fig. 4). Two patients developed prosthesis-related complications. One patient experienced prosthesis displacement and limb deformity due to an intramedullary nail locking screw fracture, requiring revision surgery with intramedullary nail replacement and internal plate fixation; the union was achieved six months after revision surgery (Fig. 5). The other patient experienced an intramedullary nail fracture 14 months after prosthesis implantation due to a slight weight-bearing incident and underwent revision surgery with intramedullary nail replacement and distal medial bone grafting, achieving healing one year postoperatively.

Regarding other complications (Table 2), two patients experienced recurrent infections at 5 and 7 months postoperatively. Both patients underwent prosthesis removal, and the previous steps of the first-stage treatment were repeated until the infection eradication criteria were met. One patient successfully recovered after prosthesis re-implantation, with no infection recurrence observed during 25 months of follow-up. The other patient experienced poor wound healing owing to repeated debridement and local flap necrosis, which ultimately resulted in amputation. Another three patients had flap-related complications. Two of them had poor wound healing and secondary soft tissue defects and underwent local pedicled axial rotation flap repair one month later, ultimately achieving successful healing. One patient developed distal partial flap necrosis 10 days postoperatively, and a hematoma was removed through debridement. The secondary defect was repaired using a thick abdominal skin graft, ultimately achieving successful healing.

**Table 2:** Complications and interventions

Cas	Complications	Time of occurrence(postoperatively)	Interventions	Time of Resolution(aftrer occurrence)
2	Prosthesis fracture	14 months	Replacement of IM and allograft	2 months
3	partial flap necrosis, pin-tract infect	10 days	Abdominal full-thickness skin graft	1 month
4	Screw fracture, prosthesis displacement	6 months	Replacement of IM and plate fixaton	6 months
5	Deep infection	5 months	IM and prosthesis removal, 7 times debridement, implant the new IM and prosthesis	4 months
6	Knee joint stiffness	/	Rehabilitation training	2 months
7	Donor site poor healing	1 months	Split-thickness skin grafts from head	1 month
8	Wound separation	10 days	Local rotation flap	1 month
10	Pin-tract infect	/	Dressing changes	2 months
13	Deep infection, flap necrosis	7 months	Amputation	29 months

At the last follow-up, none of the patients showed a recurrence of infection, and all could walk independently without crutches.

## Discussion

We evaluated 13 cases of tibial defects accompanied by soft tissue defects after debridement, which pose a significant treatment challenge. In our study, final healing was achieved in 9 out of 13 patients after an average follow-up period of 31.1 months, demonstrating the effectiveness and safety of our method for bone and soft tissue reconstruction. Traditional treatment methods have demonstrated good clinical efficacy; however, when a bone defect is large, the Ilizarov technique requires prolonged traction time, leading to an increased incidence of pin tract and soft tissue infections, nonunion of docking sites, and risk of vascular complications in the flap during traction [8, 9]. The Masquelet technique requires a large amount of autologous bone, resulting in donor-site complications such as pain, hematoma, and infection at the bone harvest site, prolonging a patient's weight-bearing time to guarantee osteogenesis [23]. In vascularized fibular grafts, anatomical mismatch leads to insufficient weight transfer before hypertrophy occurs and increases the number of surgical incisions [10, 24]. We attempted to reduce infection recurrence by thorough debridement and extended observation time and observed an infection recurrence in 2 cases (2/13), which was lower than the reported rate of 16.2% in another study [5], indicating that the application of 3D-printed prostheses did not increase the infection recurrence rate.

### Flap choice

Thorough debridement is crucial in treating infectious tibial bone defects, which may result in extensive soft tissue and bone defects. Adequate soft tissue coverage is essential for successful treatment with all bone reconstruction methods. Well-vascularized soft tissue coverage can provide physical and microbiological barriers between the bone and the external microbial flora, preventing new infections in the medullary canal [1, 9]. Additionally, good vascular conditions can effectively deliver host immune cells and antibiotics [25] and may also close dead space, promoting bone growth [10, 26]. It can also provide soft-tissue padding at the bony prominences, protecting the primary soft tissues from the pressure exerted by the bone margin. Studies report various flap options, each demonstrating a good clinical outcome. However, the flap type that yields the best clinical results is unknown [27, 28]. In our study, we preferentially considered SNFs for two reasons: 1. Elimination of vascular anastomosis. Unlike acute traumatic defects, patients with chronic infectious defects have a prolonged disease course, with chronic inflammatory stimulation and previous surgeries causing further vascular contractions, making vascular anastomosis more challenging. 2. Soft texture and abundant vascularity. The SNF is primarily nourished by the vascular bundle around the sural nerve and small saphenous vein branches, allowing the flap to be dissected without damaging major vascular pedicles [8] and ensuring maximum rotation angles. However, for the mid-to-distal tibia, the local tissue is limited, causing two patients to require fascial flaps for coverage.

In our study, patients encountered flap-related complications 4/13, higher than the reported rates of 0–28.6% [3, 7–10, 29]. Poor wound healing and necrosis may be caused by a poor local blood supply to the infected area and excessive tension on the flap edges. In the early stages of our study, the wound closure was incomplete in one patient after debridement and prosthesis implantation, necessitating additional fascia flap transplantation. Therefore, we recommend designing flaps with a diameter 2–3 cm larger than the defect area to provide sufficient space for the 3D-printed prosthesis and prevent wound closure failure owing to flap edema caused by re-elevation [13]. Additionally, for large defects (> 200 cm^2^) [30], the flap diameter can be increased appropriately. Based on our treatment experience, we applied VSD for two weeks after spacer and prosthesis implantation [31]. Negative pressure enhances flap adhesion to the spacer and prosthesis, effectively closing the subcutaneous dead space and preventing infection recurrence.

### Prosthesis design, timing of implantation, and impact on osseointegration

Effective osseointegration is a prerequisite for long-term prosthetic stability. We used a prosthesis with a porosity of 60–80% and a pore diameter of 625 ± 70 μm, observed to have a good bone ingrowth effect [22]. The prosthesis was designed using the contralateral bone structure as a mirror image.To reduce soft tissue tension, the prosthesis diameter was designed to be slightly smaller by 1 mm than the bone of the contralateral limb in order to reduce flap tension during wound closure. Moreover, a smaller diameter also promotes the forming of callus across the prosthesis and host bone.

To ensure thorough infection eradication, we extended the interval between the two treatment stages to 4 months and, in some cases, more than six months to ensure flap edge blood supply reconstruction and complete infection eradication. This approach differs from the Masquelet technique, which maintains the highest activity of the induced membrane within eight weeks. Blood supply and tension-free coverage of soft tissue are key factors for the success of 3D printing technology in bone defect repair. The application of 3D-printed prostheses allows for earlier weight bearing, providing mechanical stimulation at the bone-prosthesis interface. This could promote bone ingrowth, compensating for the reduced bioactivity of the induced membrane owing to the extended observation periods.

Our study revealed a final bone healing of 9 out of 13 patients, with an average healing time of 11.1 months. One patient exhibited callus growth across the prosthesis after a 2-year follow-up. Compared to our previous reports on femoral patients [32], callus formation was delayed, possibly owing to 1) reduced blood supply to the tibia [33] and 2) extensive soft tissue defects in all cases, which are detrimental to bone growth. Meanwhile, to promote bone ingrowth, we aimed to preserve the vertical pressure and tensile forces between the prosthesis and the bone. Therefore, we opted for the intramedullary nail to fix the prosthesis, allowing perpendicular micromovements at the bone interface and limited horizontal displacement simultaneously.

In our study, two patients ultimately developed prosthesis-related complications, both achieving healing through internal fixation replacement. Based on our previous finite element analysis [34], intramedullary nails primarily disperse stress before bone ingrowth occurs, making proper fixation of intramedullary nails crucial. One patient experienced malunion due to a proximal screw fracture, possibly related to the use of only two locking screws for proximal fixation, causing the screws to bear stress beyond their yield limit, leading to fracture. So we recommend using three screws for both proximal and distal intramedullary nail fixation to distribute stress. Another patient experienced an intramedullary nail fracture 14 months postoperatively, even though the proximal callus could be observed. We speculate that the fracture was related to the distal prosthesis design. Previous reports on 3D-printed prostheses for bone defect treatment [16, 17, 28, 35] prefer retaining more bone tissue and designing the interface of prostheses to match the host bone structure. However, uneven bone surfaces may lead to uneven stress distribution, hindering the mechanical transmission between the prostheses and bone surfaces and potentially causing metal fatigue and prosthesis fractures. Therefore, we suggested making the host bone interface sufficiently flat to ensure a uniform stress distribution during debridement osteotomy. Additionally, when defects involve the metaphysis, the inverse funnel structure and enlarged joint surface often hinder proper and stable fixation [36]. Hence, we incorporated an integrated lateral plate into the prosthesis design to ensure stability; however, this approach may affect bone ingrowth owing to stress shielding.

Our study had certain limitations. It was a retrospective study with a small sample size and lacked a comparative analysis of the outcomes between different flap types and traditional bone reconstruction methods. Longer follow-up and a large sample size are needed to corroborate the effectiveness and clinical outcomes. Additionally, this technique has the potential to limit future treatment options, making amputation one of the few outcomes available if there are bad complication.

## Conclusion

Our study demonstrated the safety and efficacy of 3D-printed porous titanium prostheses combined with flap reconstruction for treating infectious tibial bone defects. Soft tissue repair should precede bone structure repair with a prosthesis diameter slightly smaller than the original bone structure, and prosthesis implantation should be performed at least four months after flap reconstruction. We observed that applying a prosthesis did not increase the recurrence rate of infection and provided good functional recovery, offering more options for treating infectious bone defects.

## Data Availability

The datasets and raw de-identified data used and/or analysed during the current study are available from the corresponding author on reasonable request.

## Abbreviations

PMMA: Polymethylmethacrylate
VSD: Vacuum sealing drainage
WBC: White blood cell count
CRP: C-reactive protein levels
ESR: Erythrocyte sedimentation rates
SNF: Sural neurocutaneous flap
LSNF: Lesser saphenous neurocutaneous flap
